# Evaluation of mental health first aid training for family members of military veterans with a mental health condition

**DOI:** 10.1186/s12888-021-03139-9

**Published:** 2021-03-04

**Authors:** Justine Evans, Madeline Romaniuk, Rebecca Theal

**Affiliations:** 1 Gallipoli Medical Research Foundation, Greenslopes Private Hospital, Newdegate St, Greenslopes, Queensland 4120 Australia; 2 University of Queensland, St Lucia, Queensland 4072 Australia

**Keywords:** Mental health first aid, Veterans, Mental health carer, Mental health stigma, Burnout, Mental health literacy

## Abstract

**Background:**

A concerning proportion of former Australian Defence Force (ADF) members meet criteria for a mental health condition. Mental health difficulties not only affect the individual veteran. They have been found to negatively impact the mental health of family, with an increased likelihood for family members of veterans developing a mental health condition. The aim of this study was to evaluate whether participating in a Mental Health First Aid (MHFA) program improved family members of veterans mental health knowledge, reduced personal and perceived mental health stigma, reduced social distancing attitudes and increased confidence and willingness to engage in MHFA helping behaviours. Additionally, the study measured participant’s general mental health and levels of burnout.

**Method:**

The study utilised an uncontrolled design with assessment at three time points (baseline, post-program and three-month follow-up). Participants (*N* = 57) were immediate and extended family members of former ADF members with a mental health condition, who took part in a two-day standard adult MHFA program. At each time point, participants completed self-report measures assessing mental health knowledge, personal and perceived mental health stigma, social distancing attitudes, confidence and willingness to engage in MHFA helping behaviours, general mental health and burnout. Cochranes Q and repeated measures ANOVA was computed to measure the impact of time on the outcome variables.

**Results:**

Results indicated significant improvements in MHFA knowledge and confidence in providing MHFA assistance. Significant reductions in *personal* mental health stigma (i.e. an individual’s attitude towards mental health) for schizophrenia were observed and maintained at follow up. High levels of *perceived* mental health stigma (i.e. the belief an individual holds about others attitudes towards mental health) were reported with no significant changes observed following the MHFA program. Results did not indicate any significant benefit in improving general psychological distress or burnout at follow up. The participant sample had high levels of mental health difficulties with over half reporting a lifetime mental health diagnosis.

**Conclusion:**

The study is an important contribution to the international literature on MHFA. The provision of a MHFA program to family members of military veterans has not previously been evaluated. Implications of the findings are discussed with regards to future directions of MHFA research and implementing MHFA programs in this population.

## Background

A concerning proportion of military veterans experience mental health conditions. These conditions include depression, substance abuse disorder, and posttraumatic stress disorder (PTSD), and can include extreme mental distress, aggressive behaviours, or suicidality [1–3]. Van Hooff et al. [4] reported that 46.4% of ex-serving ADF members are estimated to experience a mental health disorder within a 12-month time frame. These conditions not only affect the individual ex-service personnel, but have also been found to negatively impact their family members, including spouses, children and parents [5, 6]. Family members often take on a carer role, and have an increased likelihood of developing mental health conditions themselves [5, 6]. Understanding mental health, as well as knowledge about resources and methods to access professional help can be a vital component in providing support for others as well as monitoring one’s own psychological health.

MHFA has been defined as “the help provided to a person developing a mental health problem or in a mental health crisis. The first aid is given until appropriate professional treatment is received or until the crisis resolves” [7]. MHFA is an Australian-developed standardised training program designed to address the widespread lack of knowledge surrounding mental health [8]. The standard version of the MHFA program targets mental health problems in adult populations. Numerous iterations of MHFA programs (e.g., Teen MHFA, MHFA for Aboriginal and Torres Strait Islanders MHFA for Financial Counsellors) have been developed and evaluated.

Research into the effectiveness of MHFA programs has demonstrated that participants who complete the training display significant improvements in the following areas: increased knowledge of mental health conditions (mental health literacy), a reduction in stigmatising beliefs about mental illness, and a greater likelihood of making behavioural changes (increased confidence), such as approaching a person in distress, or discussing mental health topics [8–10]. Meta-analytic findings have reported small to moderate effect sizes for improvements in mental health knowledge and small effect sizes for changes in stigma levels, confidence and increases in help-related behaviour towards others experiencing mental health difficulties [8, 11]. Hadlaczky et al. [8] found that there was no clear systematic difference in results related to the study design (i.e., with or without a control group).

Previous qualitative research has longitudinally explored the utility of MHFA programs on improving confidence and behaviours related to providing MHFA. Jorm, Kitchener, and Mugford [12] examined MHFA recipient’s subsequent behavioural experiences of providing MHFA. They found that post-training experiences of providing MHFA were reported by approximately three quarters of participants. The key theme that arose out of this research was the majority of participants had experienced a direct situation where mental health problems were identified, and the knowledge they had acquired from the MHFA program helped them to take appropriate steps to assist the person. These findings, combined with meta-analytic outcomes, provide some evidence that participation in a MHFA program can lead to behavioural outcomes, sustained improvements in mental health knowledge and changed attitudes towards mental health.

MHFA programs have been studied in varied population groups (e.g., the Australian Vietnamese community [13], the Australian Chinese Community [14], Australian adult members of the general public [15]). Within the enlisted military context, MHFA programs have been utilised in the United States Army National Guard [10] and the British Military [16]. Studies conducted with enlisted military members found a similar pattern of results to that observed in meta-analytic findings, with statistically meaningful differences in change over time for mental health literacy, reductions in stigmatising attitudes and increased confidence in providing assistance to someone experiencing mental distress [10, 16]. These results suggest that MHFA may be promising intervention for reducing barriers to engaging in help seeking, which can be particularly problematic in the military context. Van Hoof et al. [4] reported that just under a quarter of transitioned ADF personnel and regular ADF members reported never engaging with professional assistance despite being concerned about their mental health.

The literature has established a link between the experience of PTSD in ex-service personnel and the mental health of their intimate partners and immediate family members, indicating poorer psychological health [5, 17, 18]. While a spouse or family member is often an ex-service personnel’s greatest source of emotional and material support, this also has the potential to have a detrimental impact on the psychological wellbeing of family members if they feel isolated and unsupported in the caring role. A recent qualitative study that examined the experiences of spouses of ex-service personnel with PTSD identified that a barrier to support included ambivalence about the helpfulness of involving others [19, 20]. This finding suggests that providing an intervention to family members that challenges beliefs around the helpfulness of involving professional and other appropriate support could potentially assist in reducing the isolation of providing support and care to a partner or family member with mental health difficulties. While MHFA programs have been studied in a range of populations there is currently a lack of evidence regarding the efficacy of providing MHFA for family members supporting a military veteran experiencing a mental health condition.

## Methods

### Study aim

The aim of the current study was to evaluate the delivery of the standard Adult MHFA program for adult family members of military veterans with a mental health condition. To the authors knowledge this is the first evaluation that has evaluated the utility of this intervention specifically for family members supporting a military veteran with a mental health condition. The specific objectives of the current research were to investigate the impact of the standard Adult MHFA course on: (1) mental health literacy; (2) social distancing attitudes towards mental illness; (3) stigmatising attitudes (both personal and perceived) towards mental illness; (4) general psychological distress; (5) burnout; (6) confidence to engage in MHFA actions and (7) MHFA behaviours completed following the completion of the training.

### Study design

The study utilised an uncontrolled, repeated measures, longitudinal design with assessment at three time points: (1) prior to commencing the MHFA program (pre-intervention); (2) on conclusion of the MHFA program (post-intervention); and (3) three months following the conclusion of the MHFA program (follow-up).

### Participants

Participants were adult family members of an ex-serving ADF member with a mental health condition. Family members were inclusive of immediate and extended family over 18 years of age. All participants were currently residing with or in weekly contact with the ex-serving family member.

A total of 59 participants enrolled in the study. Two participants withdrew their participation prior to the second day of the MHFA program. As such, a total of 57 (51 female, 6 male) participants were included in the analysis. The participant sample was predominantly female (89.5%) with the most common relationship to an ex-serving family member being spouse (59.6%) and parent (22.8%). Key participant demographic information is presented in Table 1. Key demographic information regarding the ex-serving family members is presented in Table 2.

**Table 1:** Demographic characteristics of MHFA program participants

Demographic Variable	***(n*** = 57)
*Age* (*n* = 56)	45.3 (14.8)
*Marital Status*	
Married	38 (66.7%)
Partner/De facto	9 (15.8%)
Single	10 (17.5%)
*Lifetime mental health condition*	
No	27 (47.4%)
Yes	29 (50.9%)
Unsure	1 (1.8%)
*Reported diagnoses (n = 30):*	
PTSD	10 (33.3%)
Anxiety Disorder	13 (43.3%)
Depressive Disorder	19 (63.3%)
Panic Disorder	2 (6.7%)
Alcohol Use Disorder	2 (6.7%)
Substance Use Disorder	1 (3.3%)
Bipolar Disorder	3 (10.0%)
Other (Postnatal depression (*n* = 2); Borderline Personality	3 (10.0%)
Disorder (*n* = 1))	
≥ 2 reported diagnoses	12 (40.0%)
*Current mental health condition*	
No	39 (68.4%)
Yes	13 (22.8%)
Unsure	5 (8.8%)
*Reported diagnoses (n = 18):*	
PTSD	9 (50.0%)
Anxiety Disorder	11 (61.1%)
Depressive Disorder	12 (66.7%)
Panic Disorder	2 (11.1%)
Bipolar Disorder	2 (11.1%)
Other (Borderline Personality Disorder)	1 (5.6%)
≥ 2 reported diagnoses	8 (44.4%)
*Relationship to Ex-Service Personnel Family Member*	
Spouse of participant	34 (59.6%)
Parent of participant	13 (22.8%)
Child of participant	3 (5.3%)
Aunt/Uncle of participant	1 (1.8%)
Other (Parent in-law (n = 1); Son in-law (n = 1); Niece (*n* = 1))	3 (5.3%)
More than one family member known	3 (5.3%)

*Note.* Data presented number of participants (percent of total participants); *n* = number of participants

**Table 2:** Demographic characteristics of ex-serving family members (reported by participant)

Demographic Variable	***n*** = 57
*Age* (*n* = 53)	53.3 (14.6)
*Gender*	
Male	50 (87.7%)
Female	4 (7.0%)
More than one ex-service personnel known	3 (5.3%)
*Service type*	
Navy	3 (5.3%)
Army	40 (70.2%)
Air Force	12 (21.2%)
More than one service (Army and Air Force)	2 (3.5%)
*Medically discharged*	
No	35 (61.4%)
Yes	21 (36.8%)
Missing	1 (1.8%)
*Mental health condition* *Reported diagnoses:*	
PTSD	44 (77.2%)
Adjustment Disorder	7 (12.3%)
Anxiety Disorder	29 (50.9%)
Depressive Disorder	28 (49.1%)
Panic Disorder	7 (12.3%)
Alcohol Use Disorder	12 (21.1%)
Substance Use Disorder	2 (3.5%)
Bipolar Disorder	1 (1.8%)
Other (Acquired brain injury (n = 1); Chronic pain (n = 1); Dementia (n = 1); Phobias (n = 1); Borderline Personality Disorder and Anorexia Nervosa (n = 1)	5 (8.8%)
≥ 2 reported diagnoses	40 (70.1%)
Missing	7 (12.3%)

*Note.* Data presented as number of participants (percent of total participants); *n* = number of participants

### Ethics

Ramsay Health Care QLD Human Research Ethics Committee approved this research study. Potential participants who expressed interest in the study were provided with the Participant Information Sheet and Informed Consent Form. Written informed consent was obtained prior to any study-related activities. Study participants were family members of veterans. Participants did not have an identified mental health diagnosis. The study required that participant’s complete questionnaires regarding their psychological state and attendance at the MHFA workshop included discussion of mental health related topics. As there was potential for some discomfort to occur participants were provided with the Principle Investigator/Senior Clinical Psychologists contact details and counselling services contact details. A registered Clinical Psychologist ran the MHFA workshop.

### Procedure

Participants were recruited through veteran support and ex-service organisations (ESOs), as well as hospital and community service providers. Participants completed one of seven Standard Adult MHFA programs between August 2018 and April 2019. The MHFA programs were facilitated by an Accredited MHFA Instructor The MHFA program and all included materials are manualised to ensure standardised delivery. The materials have been developed through expert consensus by MHFA Australia in which the views of consumes, carers, and professionals are collated and synthesised.

### Description of the standard adult MHFA course

The Standard Adult MHFA course is 12 h and includes information on depression, anxiety problems, psychosis, substance use problems, and crisis situations (e.g., suicide and self-harm, panic attacks, drug/alcohol overdose). The course has a strong focus on skill building and provides education on giving MHFA using a five step action plan which describes how to practically assist individuals experiencing mental health problems or a mental health crisis. During the course, participants are taught how to apply the Action Plan to help people with mental health problems described above.

### Measures

Participants completed outcome questionnaires prior to the commencement and on immediate completion of the MHFA course, and at 3 month follow up.

#### Demographic questionnaire

Prior to commencement of the MHFA program, participants completed a demographic questionnaire which included information on: age, gender, ethnicity, employment status, marital status, number of children, educational level achieved, past and current mental health diagnosis (both participant and ex-serving family member), contact in prior 6 months with individuals experiencing mental health difficulties, and service information pertaining to the ex-serving family member.

#### Mental health first aid training effectiveness questionnaire

Participants completed the Mental Health First Aid Training Effectiveness Questionnaire at all three assessment time points. This measure assessed the followed constructs:

1. MHFA literacy;
2. Confidence to engage in MHFA actions;
3. Social distancing attitudes towards mental illness;
4. Stigmatising attitudes (both personal and perceived) towards mental illness;

MHFA Literacy was measured by asking participants to read two sequential case vignettes about a fictional person who was exhibiting symptoms of either depression (‘Mary’) or schizophrenia (‘John’) [21, 22]. For each vignette an open-ended question assessing mental health knowledge was asked: What would you say, if anything, is wrong with Mary/John? A correct score was received if depression and schizophrenia were stated in the participants’ response. To assess level of confidence in helping Mary or John participants were asked: How confident would you be in helping Mary/John*?* and responded on a 5-point likert scale from ‘Not at all’ to ‘Extremely’ confident. Overall confidence in making contact with, talking to, and providing help to a person suffering from a mental health condition was assessed on a 4-point likert scale from ‘Not at all Confident’ to ‘Very Confident’.

Additionally, as part of the MHFA Effectiveness Questionnaire, the Social Distance Scale and Stigmatic Attitudes Questionnaire were asked in the context of depression (Mary) and schizophrenia (John).

The Social Distance Scale [23], is a 5-item measure assessing social distance or avoidance behaviours directed at an individual with a mental health condition. Participants rated their willingness to interact with Mary and John on a 4-point likert scale from 1 (Definitely Willing) to 4 (Definitely Unwilling), with higher scores indicating greater social distancing or less willingness to interact with or include Mary/John. Specifically, participants rated their willingness to 1) move next door to the person in the vignette; 2) spend an evening socialising with the person; 3) make friends with the person; 4) work closely on a job with the person; and 5) have the person marry into the family. The Social Distance Scale is regularly used to assess changes in social distancing following participation in MHFA programs [21].

The Stigmatic Attitudes Questionnaire [21] is an 18-item measure assessing personal and perceived stigmatic beliefs towards individuals with a mental health condition. Level of agreement to statements based on personal stigmatising attitudes and perceived stigmatising attitudes of other people in general were rated on a 5-point likert scale from 1 (Strongly Disagree) to 5 (Strongly Agree), with higher scores indicating higher levels of stigma or greater agreement with stigmatising statements. Examples of personal stigma items are: (1) people with problems like Mary/John could snap out of it if they wanted; (2) a problem like Mary’s/John’s is a sign of personal weakness. The set of perceived stigma items covered the same statements but started with “Most other people believe”. The Stigmatic Attitudes Questionnaire has satisfactory internal consistency (α = .78–.82) and has been commonly used in evaluating the effectiveness of MHFA programs in reducing stigmatic beliefs [13, 24, 25].

#### Kessler psychological distress Scale-10 (K-10)

Participants completed the K-10 at all three assessment time points. The K-10 is a 10-item self-report scale that assesses general psychological distress based on level of nervousness, agitation, psychological fatigue, and depression in past 30 days. Questions on emotional states are rated on a 5-point Likert scale from 1 (None of the time) to 5 (All of the time), with higher scores indicating higher psychological distress. A total score of 30 or more is associated with a severe mental disorder. The K-10 has demonstrated strong psychometric properties and is regularly used to assess psychological distress and predict severe mental illness in the general population [26].

#### Burnout measure – short version (BMS)

Participants completed the BMS at all three assessment time points. The BMS is a 10-item self-report measure that assesses level of burnout based on physical, emotional, and mental exhaustion [27]. The BMS has been modified for this study to replace ‘work overall’ with ‘supporting your veteran family member’ to assess burnout related to caring for a family member with a mental health condition. Questions are rated on 7-item likert scale from 1 (Never) to 7 (Always), with higher scores indicating higher levels of burnout (range = 10 to 70). A mean score of 3.5 or more is indicative of burnout. Research indicates the BMS is a valid and reliable measure with satisfactory internal consistency (α = .85–.92) and test-retest reliability.

#### Post program questionnaire

On immediate completion of the MHFA program participants completed the Post-Program Questionnaire. This included a four-item measure assessing attitudes towards mental health [28]. Agreement with items was rated on a 5-point likert scale from “Strongly Disagree” to “Strongly Agree”. Additionally, the Post-Program Questionnaire assessed the acceptability of the MHFA program through participant feedback regarding their experiences and satisfaction with the program.

#### Follow-up questionnaire

The Follow-up Questionnaire included a 6-item measure adapted from Mendenhall and colleagues [28] assessing skills gained, retained, and utilised from the MHFA program. Items were rated on a 3-point scale (Yes, No, Unsure). Additionally, the Follow-up Questionnaire assessed post course experiences in applying skills learned from the MHFA training course, adapted from a qualitative interview designed by Jorm and colleagues [12]. Participants were asked to describe a post-course situation where someone seemed to have a mental health problem and if they did anything specific to help the person. Alternately, for those who did not experience a post-course situation, participants were asked to discuss their expectations and level of confidence with such a situation if it arose in the future.

### Statistical analysis

Statistical analysis was conducted using IBM Statistical Package for Social Sciences (SPSS v26). Continuous data was reported as means (standard deviation). Categorical data was reported as number of participants (percent of total participants). Independent samples t-tests and Fishers exact tests were used to compare differences in outcome measures at pre-intervention in participants who did and did not complete follow up measures. One-way repeated measures analysis of variance (ANOVA) were conducted to determine if continuous outcome measures were significantly different across time points. Differences between pre-intervention, post-intervention, and follow-up outcomes were considered significant at *p* ≤ 0.05. Post-hoc analysis was conducted using the Bonferroni correction. Effect sizes were calculated using multivariate partial eta squared and were considered large when greater than 0.14. Assumptions for one-way repeated ANOVAs were met. Cochran Q tests were conducted to determine if binomial categorical data outcomes were significantly different across time points. If sufficient sample size was not available, an exact *p*-value was utilised. All other assumptions for Cochran Q tests were met. Differences between pre-intervention, post-intervention, and follow-up outcomes were considered significant at *p* ≤ 0.05. Post-hoc analysis was conducted using McNemar’s test, followed by Bonferroni correction. Post-hoc differences were considered significant at *p* ≤ 0.0167.

## Results

### Participation and response rates

Two participants withdrew from the study following consent. Follow up response rate was 68.4% (*n* = 39). As displayed in Table 3 there were significant differences in outcome measures at pre-intervention between participants who did and did not complete follow up questionnaires. Participants who did not complete the follow up had significantly lower confidence in helping Mary (someone with depression) and increased perceived stigma towards people with depression. Additionally, there was a trend towards increased personal stigma towards people with schizophrenia and increased burnout in participants who did not complete the follow up compared to those who did. There were no significant differences between age, gender or current mental illness between groups.

**Table 3:** Pre-intervention differences between participants who did and did not complete follow-up

Demographic Variable	Follow-up completed	No Follow-up	***P-***value
Age	43.5 (14.0.23) (*n* = 39)	49. (16.2) (*n* = 18)	.180
Gender (% Female)	36 (92.3%) (*n* = 39)	15 (83.3%) (*n* = 18)	.368
Current Mental Health Condition (% Yes)	11 (28.2%) (*n* = 39)	2 (11.1%) (*n* = 18)	.228
Confidence			
Helping Mary (% quite a bit)	16 (41.0%) (*n* = 39)	2 (11.1%) (*n* = 18)	.032*
Helping John (% quite a bit)	12 (30.8%) (*n* = 39)	2 (11.1%) (*n* = 18)	.099
Social Distance			
Depression	8.90 (2.92) (*n* = 39)	8.89 (2.54) (*n* = 18)	.992
Schizophrenia	10.5 (3.41) (*n* = 39)	9.94 (3.80) (*n* = 18)	.610
Stigmatic Attitudes Questionnaire – Depression			
Personal Stigma	14.4 (3.19) (*n* = 37)	16.4 (4.78) (*n* = 18)	.069
Perceived Stigma	31.4 (4.71) (*n* = 38)	35.3 (3.77) (*n* = 17)	.004*
Stigmatic Attitudes Questionnaire - Schizophrenia			
Personal Stigma	18.4 (3.72) (*n* = 39)	20.9 (5.85) (*n* = 17)	..051
Perceived Stigma	33.7 (5.96) (*n* = 39)	35.8 (5.14) (*n* = 18)	.190
K10 Distress Scale			
Total Score	17.7 (6.57) (*n* = 39)	17.9 (5.23) (*n* = 18)	.875
Severity	Likely to be well	Likely to be well	
Burnout Measure			
Mean Score	3.25 (1.27) (*n* = 38)	3.87 (0.78) (*n* = 17)	.071
Severity	Danger signs of burnout	Burnout	

*Note.* Data presented as mean ± standard deviation or number of participants (percent of total participants); *n* = number of participants

### Mental health first aid literacy

The proportion of participants who correctly identified depression and schizophrenia at all three time points, with Cochran’s Q Test and post-hoc pairwise comparisons results, are reported in Table 4. Cochran’s Q test determined that there was no statistically significant difference in the proportion of participants who identified depression correctly over time (χ^2^(2) = 2.33, *p* = 0.311, *n* = 38). Depression –related MHFA literacy was high at pre-intervention (91.2% of all participants correctly identified) and did not significantly change across time points. Cochran’s Q test determined that there was a statistically significant difference in the proportion of participants who identified schizophrenia correctly over time (χ^2^(2) = 16.5, *p* < 0.001, *n* = 36). There was a significant increase in the proportion of participants who correctly identified schizophrenia between pre and post intervention and pre-intervention and follow up.

**Table 4:** Changes in Mental Health First Aid Literacy and Confidence for Cochran’s Q by Time

Outcome	T1		T2		T3		***P*** Value	Pair Differences by Time		
	*n*	*%* *(95% CI)*	*n*	*%* *(95% CI)*	*n*	*%* *(95% CI)*	*p*	T1 to T2	T2 to T3	T1 to T3
**Mental Health First Aid Literacy**										
Depression correctly	36	94.7 (89.5–100)	33	86.8 (78.9–94.7)	34	89.5 (81,6–97.4)	.311^a^	–	–	–
identified (*n* = 38)										
Schizophrenia correctly	16	48.5 (33.3–61.1)	30	90.9(86.7–100)	28	84.8(76,7093,3)	<.001**	<.001***	–	<.001***
identified (*n* = 33)										
**Mental Health First Aid Confidence (*n*** **(%) ≥ quite a bit confident)**										
Helping Mary	16	41.0(28.2–53.8)	35	89.7 (82.1–97.4)	34	87.2 (79.5–94.9)	<.001***	<.001***	–	<.001***
(Depression) (*n* = 39)										
Helping John	10	27.7 (16.7–41.7)	24	66.7 (52.8–77.8)	17	47.2(33,3–61.1	.001***	<.001***	–	–
(Schizophrenia) (*n* = 36)										

*Note:* Data presented as number of participants,percent of total participants (95% confidence interval); *n* Number of participants, *CI* Confidence interval; Post hoc tests (McNemar’s tests) using the Bonferroni correction by time with required significance level of *p* < .0167; T1 = Pre-intervention; T2 = Post-intervention; T3 = Follow-up; ^a^ Exact *p-*value *Significant at *p* < .05 **Significant at *p* < .01 level *** Significant at *p* < .001

### Mental health first aid confidence

There was statistically significant difference in the proportion of participants who reported high confidence levels (quite a bit or extremely confident) in helping a person with depression (χ^2^(2) = 34.3, *p* < 0.001, *n* = 39) and schizophrenia over time (χ^2^(2) = 14.0, *p* = 0.001, *n* = 36). There was a significant difference in reported confidence in helping someone with depression between pre and post intervention and pre-intervention and follow up. There was a significant difference in reported confidence in helping someone with schizophrenia between pre and post intervention. However, this was not maintained at follow-up (see Table 4).

Table 5 reports the result of a series of one-way repeated measures ANOVA on MHFA program outcomes. A series of ANOVA analyses were conducted to compare overall confidence scores for making contact with (Wilks’ Lambda = 0.677, *F*(2,34) = 8.09, *p* < 0.001, multivariate partial eta squared = 0.323), speaking to (Wilks’ Lambda = 0.635, F(2,35) = 10.1, *p* < 0.001, multivariate partial eta squared = 0.365), helping someone with a mental health condition (Wilks’ Lambda = 0.491, *F*(2,35) = 18.1, *p* < 0.001, multivariate partial eta squared = 0.509), and total confidence (Wilks’ Lambda = 0.541, *F*(2,35) = 14.8, *p* < 0.001, multivariate partial eta squared = 0.459) at all time points. There was significant effect for time for making contact with someone with a mental health condition, talking to someone with a mental health condition, helping someone with mental health condition, and total confidence scores. Post-hoc analysis revealed that all confidence scores, including total confidence scores, significantly increased between pre and post intervention and were maintained at follow-up (see Table 5).

**Table 5:** Means, standard deviations, and significance values for one-way ANOVAs by Time for MHFA program outcomes

Outcome	T1		T2		T3		ANOVA significance	Pair Differences by Time (***p***)		
	*M*	*SD*	*M*	*SD*	*M*	*SD*	*p*	T1 to T2	T2 to T3	T1 to T3
**Mental Health First Aid Confidence**										
Making Contact (*n* = 36)	2.78	0.87	3.33	0.79	3.47	0.56	<.001***	.006**	–	.001***
Talking (*n* = 37)	2.95	0.71	3.51	0.61	3.54	0.56	<.001***	<.001***	–	.001***
Providing Help (*n* = 37)	2.59	0.83	3.43	0.69	3.46	0.65	<.001***	<.001***	–	<.001***
Total Score (*n* = 37)	8.24	2.22	10.3	1.92	10.5	1.52	<.001***	<.001***	–	<.001***
**Social Distancing Total**										
Depression (*n* = 39)	8.90	2.92	7.67	2.63	7.92	3.22	.004**	.003**	–	–
Schizophrenia (*n* = 37)	10.4	3.33	8.41	3.03	9.32	3.67	<.001***	<.001***	–	–
**Stigmatic Attitudes Total**										
Personal (Depression) (*n* = 37)	14.4	3.19	12.2	3.74	12.9	2.90	<.001***	<.001***	–	–
Perceived (Depression) (*n* = 38)	31.4	4.71	31.1	5.84	32.0	6.17	0.693	–	–	–
Personal (Schizophrenia) (*n* = 37)	18.3	3.79	16.4	6.11	16.0	4.62	.003**	–	–	.002**
Perceived (Schizophrenia) (*n* = 37)	43.2	5.50	34.7	6.22	34.1	6.44	0.847	–	–	–
**Psychological Distress (K-10)** (*n* = 37)	17.4	6.56	20.2	7.51	17.4	7.50	0.017*	.012*	–	–
**Burnout** (*n* = 36)	3.20	1.28	2.96	1.32	3.27	1.36	0.088	–	–	–

*Note:* Data presented as means and standard deviations; *n* = number of participants; Post hoc tests using the Bonferroni correction by time; T1 = Pre-intervention; T2 = Post-intervention; T3 = Follow-up; *Significant at *p* < .05 **Significant at *p* < .01 level *** Significant at *p* < .001

### Social distancing

A one-way repeated measures ANOVA was conducted to compare scores on the Social Distance Scale (for depression and schizophrenia) at all time points. There was significant effect for time for depression (Wilks’ Lambda = 0.743, *F*(2,37) = 6.40, *p* = 0.004, multivariate partial eta squared = 0.257) and schizophrenia (Wilks’ Lambda = 0.608, *F*(2,35) = 11.3, *p* < 0.001, multivariate partial eta squared = 0.392). Post-hoc analysis revealed that Social Distance Scale score for depression and schizophrenia significantly decreased from pre-intervention to post-intervention, indicating a reduction in unwillingness to interact with someone with symptoms of depression and psychosis (see Table 5). However, this was not maintained at follow-up.

### Personal and perceived stigmatic attitudes

A one-way repeated measures ANOVA was conducted to compare scores on the Stigmatic Attitudes Questionnaire (for depression) at all time points. There was significant effect for time for personal stigma (Wilks’ Lambda = 0.550, *F*(2,35) = 14.3, *p* < 0.001, multivariate partial eta squared = 0.450). There was no significant effect for time for perceived stigma (Wilks’ Lambda = 0.980, *F*(2,36) = 0.37, *p* = 0.693, multivariate partial eta squared = 0.020). Post-hoc analysis revealed that personal stigma scores on the Stigmatic Attitudes Questionnaire for depression significantly decreased from pre-intervention to post-intervention. However, this was not maintained at follow up (see Table 5).

A one-way repeated measures ANOVA was conducted to compare scores on the Stigmatic Attitudes Questionnaire (for schizophrenia) at all time points. There was significant effect for time for personal stigma (Wilks’ Lambda = 0.711, F(2,35) = 7.11, *p* = 0.003, multivariate partial eta squared = 0.289). There was no significant effect for time for perceived stigma (Wilks’ Lambda = 0.991, *F*(2,35) = 0.166, *p* = 0.847, multivariate partial eta squared = 0.009). Post-hoc analysis revealed that personal stigma scores on the Stigmatic Attitudes Questionnaire for schizophrenia significantly decreased from pre-intervention to follow-up (see Table 5).

### General psychological distress

A one-way repeated measures ANOVA was conducted to compare scores on the K-10 at all time points. There was significant effect for time for general psychological distress (Wilks’ Lambda = 0.791, *F*(2,35) = 4.62, *p* = 0.017, multivariate partial eta squared = 0.209). Post-hoc analysis revealed that general psychological distress significantly increased from pre-intervention to post-intervention. However, this was not maintained at follow up. Results indicate that psychological distress returned to pre-intervention levels at follow-up (see Table 5). Cut-off scores from the 2001 Victorian Population Health Survey indicate participants are ‘likely to be well’ at all time points.

### Burnout

A one-way repeated measures ANOVA was conducted to compare scores on the Burnout Measure at all time points. There was no significant effect for time indicating burnout scores were not significantly different at any time point (Wilks’ Lambda = 0.867, *F*(2,34) = 2.61, *p* = 0.088, multivariate partial eta squared = 0.133). Mean scores on the Burnout Measure indicated participants were showing ‘danger signs of burnout’ at all time points.

### Recall and use of MHFA skills

At follow-up, 53.8% of participants (*n* = 21) reported having experienced a post-course situation where someone seemed to be having a mental health-related issue. Post-course situations included experiences with depression, anxiety, PTSD, panic attack, bipolar mania, suicidal thoughts, self-harm, and drug and alcohol use. Of those who had experienced a post-course situation, 90.5% (*n* = 19) reported being able to provide help or support to those dealing with a mental-health related issue and believed their support made a positive impact. For those who did not experience a post-course situation, 93.7% (*n* = 15) reported feeling sufficiently prepared or confident to deal with someone believed to be suffering from a mental health problem.

## Discussion

This study aimed to evaluate the effectiveness of providing the Standard Adult MHFA program to adult family members of ex-service personnel with a mental health condition, with a specific focus on the impact of the MHFA program on mental health knowledge, stigmatising and social distancing attitudes, and confidence and willingness to engage in helping behaviour.

There was a significant increase in participant’s mental health knowledge related to psychotic disorders. This is in line with previous findings of small to moderate effect sizes for improvements in mental health knowledge following completion of a MHFA program [8, 11]. There was a ceiling effect for increases in knowledge related to depression, as the percentage of people able to recognise depression before participating in the MHFA program was above 90%. The pre-course recognition scores were higher than an Australian national survey of adults, where the percentage of adults able to correctly identify depression has been reported at 73.7% [29]. This may be attributable to the study population, who were exposed to a partner or family member with a chronic mental health condition. This population is potentially likely to have higher rates of mental health knowledge and ability to recognise mental health conditions in others.

Consistent with prior research the results indicated that personal stigma was higher for psychosis (schizophrenia) than depression [21]. However, the current sample did not overall hold highly stigmatising (personal) attitudes toward others, particularly for depression. Despite this, personal stigmatic attitudes towards depression did significantly reduce at post intervention. For schizophrenia, results indicated that personal stigmatic attitudes towards this disorder were significantly reduced at follow up. The magnitude of the effect size for reductions in personal stigma towards schizophrenia was large, in comparison to previous meta-analyses that have reported a small effect size for reductions in stigma [8, 11].

Beliefs about perceived stigma by the wider community was greater than personal stigma, with no significant attitudinal changes to perceived stigma between pre- and post-intervention, and at follow up. Levels of personal stigma towards individuals with a mental health condition were significantly less than perceived stigma at all time points for both depression and schizophrenia. Participants rated high levels of perceived stigma, particularly for schizophrenia. Griffiths et al. [21] found overall perceived stigma in the general Australian population was substantial. It is also relevant to note that in the military context family members of participants may have experienced negative consequences due to their mental health condition (i.e., 36.8% of family members were medically discharged).

There were significant improvements in levels of confidence in providing assistance to a person with depression (which included confidence in making contact, talking to and providing help to the person) and this was maintained at follow up. While there were significant improvements in levels of confidence in providing assistance to a person with schizophrenia at post-intervention, these improvements were not maintained at follow up. Given the low base rates of schizophrenia in the population (approximately 1 in 200 individuals) it is possible that participants may experience lower levels of confidence in assisting someone with this condition due to lack of exposure or contact. Results indicated that overall confidence in making contact with, talking to and helping someone with a mental health condition improved at post-intervention and this was maintained at follow up. The magnitude of the effect size for increases in overall confidence was large, in comparison to previous meta-analyses which have reported a small effect size for increases in confidence [8, 11].

The actual provision of MHFA following the program was reported by approximately half the participants who participated in the follow up. Of the participants who had experienced a post-course situation, 90% reported being able to provide help or support to those dealing with the mental health issue and believed that their support had made a positive impact. Hadlaczky et al. [8] found a small effect size for changes in help related behaviour. Morgan et al. [11] found that there were small improvements in the actual help provided by MHFA recipients to a person with a mental health problem but that the quality of behaviours provided was unclear.

There was a significant decrease in social distancing attitudes from pre- to post-intervention (for both depression and schizophrenia) which suggests an increased willingness to be close to someone with these mental health conditions. However, this finding was not maintained at follow up. It is important to put this finding into the context of low reported levels of social distancing reported by participants prior to engaging in the MHFA program. Given that participants were family members of a person with a mental health condition, with the most common relationship being spouse or parent, it is not surprising that there were low levels of social distancing attitudes in the sample. Similar to the results found for personal and perceived stigma, participants expressed more negative social distancing attitudes towards schizophrenia than depression. This finding is consistent with prior literature [21].

General psychological distress was found to have significantly increased at post-intervention but returned to pre-intervention levels at follow-up. There have been mixed previous findings regarding the impact of MHFA programs on participant mental health. Morgan et al. [11] identified that findings regarding the impact of MHFA on recipients mental health were unclear, with potentially very small positive effects at six monthly follow up. The finding of increased distress post-intervention (but returning to the normal range at 3 month follow up) provides further data on the potential impact of MHFA programs on psychological distress. The program does not target improving mental health, and it may have been in the current sample (given significant rates of lifetime mental health difficulties and elevated levels of burnout) that focusing on discussions around mental health brought with it difficult emotions and distress. The finding of high levels of self-reported mental health difficulties is consistent with prior literature that has established a link between the experience of PTSD in ex-service personnel and poorer mental health of their intimate partners and immediate family members [5, 17, 18, 30].

Burnout was a novel outcome investigated in the study that has not been explored in previous MHFA trials. The BMS assessed burnout based on physical, emotional and mental exhaustion. In this cohort, burnout levels were sub-threshold (indicating danger signs) for burnout at all assessment time points. Results indicated that although levels of burnout decreased slightly at post-intervention, there was no significant improvement to burnout scores at post-intervention or follow up. The elevated levels of lifetime mental health difficulties and burnout in this population suggests that it would be beneficial to adapt the standard 12-h Adult MHFA course to include the impact of having a family member with a chronic mental health condition on the mental health of family members. Both of the previous MHFA interventions run with military groups have been adapted to the context and included information specific to serving members [10, 16].

Despite the strengths of the current research study, it is important to consider the limitations associated with the research design and interpretation of outcomes. The sample was predominantly female partners of ex-service personnel, which may limit the generalisability of the data to other familial relationships. Partners of ex-service personnel with a mental health condition have been shown to exhibit increased risk of mental health difficulties [5, 17, 18]. It is possible that family members who are not the primary source of support for the ex-service personnel may not experience the high rates of mental health diagnosis observed in this sample. Additionally, the lack of control group limits the confidence in which the findings can be attributed to the MHFA program. It is important to note however, that a meta-analysis of MHFA programs found that uncontrolled trials produced similar effect sizes to controlled trials [8]. In this meta-analysis, nine of the fifteen selected papers were single group, pre/post studies. Morgan et al. [11] used more stringent criteria (to reduce the possibility of overestimating intervention effects) and identified similar trends in the data. This may suggest that while the current study cannot confidently attribute outcomes to the MHFA program, the outcomes observed may not be an unduly biased estimate of the intervention effect.

Furthermore, an additional limitation of the current study is the measurement of behavioural change. Intention to provide MHFA is measured with follow up data assessing whether participants have provided a specific incidence of MHFA, but there is no standardised measurement rating system in the literature to assess the quality of MHFA provided following attending a MHFA program [11]. As a consequence, it can be difficult to determine the impact of the MHFA program on MHFA skills applied in specific situations. The follow-up response rate was 68.4% with evidence that the participants ‘lost to follow-up’ had significantly lower confidence in supporting someone with a mental health condition prior to the MHFA training. This limits the interpretability of the findings, and future research should aim to address participant retention. Further, it is important to note that analysis was based on complete cases and as such, impact on program outcomes for the total sample (i.e. including those lost to follow-up) is unknown and the analysis design suggests that results are not generalisable to other populations.

These limitations point to a number of future directions for research evaluating the effectiveness of MHFA in family members of veterans. Future research may proceed in a number of ways to improve evidence-base for this population and address limitations. This may include conducting a waitlist controlled trial of the MHFA program within an organisational context that has the capacity to generate enough participant referrals to make use of an existing wait list. A controlled trial can provide evidence for efficacy of the standard Adult MHFA program for family members of ex-service personnel. Additionally, incorporating a mixed methods approach to evaluating the quality of MHFA actions provided following the completion of a MHFA program. This may help determine if intentions predict first aid actions, and assist in developing a coding system to assess the quality of MHFA actions provided. Finally, exploration of improvement of both the veteran and the partner in terms of willingness to seek support and the influence of the partner’s knowledge on the outcome of the veteran would be of use.

## Conclusions

The evaluation found increased participant mental health knowledge, increased overall confidence in providing assistance to someone with a mental health condition and reductions in personal stigma. The mean scores for mental health knowledge, stigma and social distancing attitudes reflected that participants entered the program with a good knowledge of mental health and did not view those with a mental health condition as negatively as the broader Australian population. There were no significant attitudinal changes related to perceived stigma (beliefs about stigma that others hold about mental illness). Provision of MHFA was reported by approximately half of those that participated in the 3 month follow up, with 90% of these participants reported the belief that their support had made a positive impact.

The current study is an important contribution to the international literature on MHFA, through evaluating the efficacy of implementing the Standard Adult MHFA program with family members of ex-service personnel with a mental health condition. This population has not previously been evaluated in a peer-reviewed publication and warrants further investigation given the unique stressors and mental health challenges this group experience.

## Data Availability

The datasets generated and/or analysed during the current study are not publicly available due to the data for this study including sensitive personal clinical information, and as such does not have ethical approval by the RHREC or the DDVA HREC to be made available by data deposition. However, if a reviewer or researcher was interested in obtaining the data to confirm the findings and/or to conduct analysis, then a specific request to obtain the data could be made and approved (if ethically sound) by the RHREC and the DDVA HREC. Requests must be made in writing to all of the following personnel: Dr. Madeline Romaniuk, Principal Investigator, Gallipoli Medical Research Institute, Greenslopes Private Hospital, Newdegate Street, Greenslopes, QLD 4120, AUSTRALIA; Professor Darrell Crawford, Director of Research, Gallipoli Medical Research Institute, Greenslopes Private Hospital, Newdegate Street, Greenslopes, QLD 4120, AUSTRALIA; Mr. Ian Tindall, Chair of the Departments of Defence and Veterans’ Affairs Human Research Ethics Committee, CP3–6-037, PO Box 7911, Department of Defence, Canberra, ACT 2610, AUSTRALIA.

## Abbreviations

ADF: Australian Defence Force
ANOVA: Analysis of Variance
BMS: Burnout Measure – Short Version
ESOs: Ex-Service Organisations
GPH: Greenslopes Private Hospital
K-10: Kessler Psychological Distress Scale-10
MHFA: Mental Health First Aid
PTSD: Post Traumatic Stress Disorder
SPSS: Statistical Package for Social Sciences

## References

[1] Hodson S, McFarlane A (2016). Australian veterans: identification of mental health issues. Royal Austr Coll Gen Pract.

[2] Scarr E. Changing attitudes to mental illness in the Australian Defence Force: A long way to go … . : Department of Parliamentary Services: Australian Parliamentary Library; 2015. Available from: https://www.aph.gov.au/About_Parliament/Parliamentary_Departments/Parliamentary_Library/pubs/APF/monographs/changing_attitudes.

[3] Krysinska K, Lester D (2010). Post-traumatic stress disorder and suicide risk: a systematic review. Arch Suicide Res.

[4] Van Hooff M, Lawrence-Wood E, Hodson S, Sadler N, Benassi H, Hansen C (2018). Mental health prevalence, mental health and wellbeing study.

[5] Dekel R, Monson CM (2010). Military-related post-traumatic stress disorder and family relations: current knowledge and future directions. Aggress Violent Behav.

[6] O'Toole BI, Outram S, Catts SV, Pierse KR (2010). The mental health of partners of Australian Vietnam veterans three decades after the war and its relation to veteran military service, combat, and PTSD. J Nerv.

[7] Kitchener BA, Jorm AF, Kelly C (2017). Mental health first aid manual.

[8] Hadlaczky G, Hökby S, Mkrtchian A, Carli V, Wasserman D (2014). Mental health first aid is an effective public health intervention for improving knowledge, attitudes, and behaviour: a meta-analysis. Int Rev Psychiatry.

[9] Jorm AF, Kitchener BA, Sawyer MG, Scales H, Cvetkovski S (2010). Mental health first aid training for high school teachers: a cluster randomized trial. BMC Psychiatry.

[10] Mohatt NV, Boeckmann R, Winkel N, Mohatt DF, Shore J (2017). Military mental health first aid: development and preliminary efficacy of a community training for improving knowledge, attitudes, and helping behaviors. Mil Med.

[11] Morgan AJ, Ross A, Reavley NJ (2018). Systematic review and meta-analysis of mental health first aid training: effects on knowledge, stigma, and helping behaviour. PLoS One.

[12] Jorm AF, Kitchener BA, Mugford SK (2005). Experiences in applying skills learned in a mental health first aid training course: a qualitative study of participants' stories. BMC Psychiatry.

[13] Minas H, Colucci E, Jorm AF (2009). Evaluation of mental health first aid training with members of the Vietnamese community in Melbourne, Australia. Int J Ment Health Syst.

[14] Lam AY, Jorm AF, Wong DF (2010). Mental health first aid training for the Chinese community in Melbourne, Australia: effects on knowledge about and attitudes toward people with mental illness. Int J Ment Health Syst.

[15] Jorm AF, Kitchener BA, Fischer JA, Cvetkovski S (2010). Mental health first aid training by e-learning: a randomized controlled trial. Aust N Z J Psychiatry.

[16] Crone D, Sarkar M, Loughren E, Curran T, Baker C, Hill D (2016). Evaluation of mental health first aid in the armed forces community project: final report.

[17] Calhoun PS, Beckham JC, Bosworth HB (2002). Caregiver burden and psychological distress in partners of veterans with chronic posttraumatic stress disorder. J Trauma Stress.

[18] Taft CT, Watkins LE, Stafford J, Street AE, Monson CM (2011). Posttraumatic stress disorder and intimate relationship problems: a meta-analysis. J Consult Clin Psychol.

[19] Dekel R, Goldblatt H, Keidar M, Solomon Z, Polliack M (2005). Being a wife of a veteran with posttraumatic stress disorder. J Fam.

[20] Murphy D, Palmer E, Hill K, Ashwick R, Busuttil W (2017). Living alongside military PTSD: a qualitative study of female partners' experiences with UK veterans. J Mil Veteran Fam Health.

[21] Griffiths KM, Nakane Y, Christensen H, Yoshioka K, Jorm AF, Nakane H (2006). Stigma in response to mental disorders: a comparison of Australia and Japan. BMC Psychiatry.

[22] Yap M, Jorm A (2012). Young people's mental health first aid intentions and beliefs prospectively predict their actions: findings from an Australian National Survey of youth. Psychiatry Res.

[23] Link BG, Phelan JC, Bresnahan M, Stueve A, Pescosolido BA (1999). Public conceptions of mental illness: labels, causes, dangerousness, and social distance. Am J Public Health.

[24] Burns S, Crawford G, Hallett J, Hunt K, Chih HJ, Tilley PJM (2017). What’s wrong with John? A randomised controlled trial of mental health first aid (MHFA) training with nursing students. BMC Psychiatry.

[25] Jensen KB, Morthorst BR, Vendsborg PB, Hjorthøj C, Nordentoft M (2016). Effectiveness of mental health first aid training in Denmark: a randomized trial in waitlist design. Soc Psychiatry Psychiatr Epidemiol.

[26] Furukawa TA, Kessler RC, Slade T, Andrews G (2003). The performance of the K6 and K10 screening scales for psychological distress in the Australian National Survey of mental health and well-being. Psychol Med.

[27] Malach-Pines A (2005). The burnout measure, short version. Int J Stress Manag.

[28] Mendenhall AN, Jackson SC, Hase S (2013). Mental health first aid USA in a rural community: perceived impact on knowledge, attitudes, and behavior. Soc Work Ment Health.

[29] Reavley NJ, Jorm AF (2011). Recognition of mental disorders and beliefs about treatment and outcome: findings from an Australian national survey of mental health literacy and stigma. Austr NZ J Psych.

[30] Nuwara AS, Masa'Deh R, Hamdan-Mansour AM, Qhah IK (2019). Risk of posttraumatic stress disorder and its relationship with perceived social support among family caregivers of individuals with schizophrenia or bipolar disorder. J Psychosoc Nurs Ment Health Serv.

